# Effect of CGRP and sumatriptan on the BOLD response in visual cortex

**DOI:** 10.1007/s10194-011-0415-4

**Published:** 2012-01-14

**Authors:** Mohammad S. Asghar, Adam E. Hansen, Henrik B. W. Larsson, Jes Olesen, Messoud Ashina

**Affiliations:** 1 Danish Headache Center and Department of Neurology, Faculty of Health Sciences, Glostrup Hospital, University of Copenhagen, Nordre Ringvej 57, Glostrup, 2600 Copenhagen, Denmark; 2 Functional Imaging Unit and Department of Radiology, Faculty of Health Sciences, Glostrup Hospital, University of Copenhagen, Copenhagen, Denmark; 3 Functional Imaging Unit and Department of Clinical Physiology and Nuclear Medicine, Faculty of Health Sciences, Glostrup Hospital, University of Copenhagen, Copenhagen, Denmark

**Keywords:** Calcitonin gene-related peptide (CGRP), Headache, Brain activity, Functional MRI, Sumatriptan, BOLD

## Abstract

To test the hypothesis that calcitonin gene-related peptide (CGRP) modulates brain activity, we investigated the effect of intravenous CGRP on brain activity in response to a visual stimulus. In addition, we examined if possible alteration in brain activity was reversed by the anti-migraine drug sumatriptan. Eighteen healthy volunteers were randomly allocated to receive CGRP infusion (1.5 μg/min for 20 min) or placebo. In vivo activity in the visual cortex was recorded before, during and after infusion and after 6 mg subcutaneous sumatriptan by functional magnetic resonance imaging (3 T). 77% of the participants reported headache after CGRP. We found no changes in brain activity after CGRP (*P* = 0.12) or after placebo (*P* = 0.41). Sumatriptan did not affect brain activity after CGRP (*P* = 0.71) or after placebo (*P* = 0.98). Systemic CGRP or sumatriptan has no direct effects on the BOLD activity in visual cortex. This suggests that in healthy volunteers both CGRP and sumatriptan may exert their actions outside of the blood–brain barrier.

**Electronic supplementary material:**

The online version of this article (doi:10.1007/s10194-011-0415-4) contains supplementary material, which is available to authorized users.

## Introduction

Over the past 20 years, calcitonin gene-related peptide (CGRP) has become a major focus of headache research [1]. CGRP has a wide distribution throughout the central and peripheral nervous systems [2]. The headache-related pharmacological effects of CGRP were initially studied in the peripheral nervous system, in particular, in the perivascular space [3–5]. The first in vitro study demonstrated that CGRP is spontaneously released by cultured trigeminal ganglion cells, and CGRP-containing nerve fibers form a dense network around cerebral vessels originating in the trigeminal ganglia [6]. Goadsby et al. reported the first human evidence of CGRP release in the cranial circulation after thermocoagulation [7] and CGRP infusion in patients provokes migraine attacks [8, 9]. Efficacy of CGRP receptor antagonists [10, 11] in the acute treatment of migraine finally proved the crucial role of CGRP in migraine and stimulated the interest in its mechanisms of action. The most crucial question is perhaps whether CGRP and its antagonists act in the peripheral or central nervous system [12]. It has recently been suggested that CGRP, in addition to its strong vasodilatory effect, also acts as an important and widespread neuromodulator in the brain [9, 12–14].

Using high resolution MRI angiography, we recently reported that exogenous CGRP dilates extracranial arteries and that this effect was blocked by anti-migraine drug sumatriptan [14]. Whether exogenous CGRP also affects neuronal activity in the trigeminal pain pathways in man is unknown. Functional MRI (fMRI) using the blood oxygenation level-dependent (BOLD) response is the most commonly employed method for in vivo studies of activity in the human brain [15, 16]. The BOLD response is an indirect method that measures neuronal activity by recording associated changes in cerebral hemodynamics [15–17]. The present study was primarily designed to test the hypothesis that exogenous CGRP and sumatriptan affect the BOLD response. We used a reversed checkerboard visual stimulation because it is a well validated, reproducible stimulation modality known to produce a large BOLD signal [18]. The effect of intravenous infusion of CGRP and subcutaneous injection of sumatriptan on the BOLD response was investigated in a placebo-controlled, randomized, double-blind, crossover study of normal volunteers. We hypothesized that CGRP infusion would alter the BOLD response in the visual cortex and that the selective anti-migraine drug, a 5-HT (types 5-HT1D and 5-HT1B) agonist sumatriptan would reverse CGRP induced alterations in the BOLD response.

## Methods

### Subjects

We recruited 18 healthy volunteers [11 F and 7 M; mean age 25 years (range 22–28) and mean weight 65 kg (range 53–77 kg]. Exclusion criteria were a history of serious somatic disease, migraine or any other type of headache (except episodic tension-type headache less than once a month), daily intake of any medication except contraceptives; and contraindications for MRI scan.

All female participants used safe contraceptive methods.

### Standard protocol approval, trail registration, and patient consents

All participants gave informed consent to participate. The Ethical Committee of Copenhagen (H-KA-20060083) approved the study, which was conducted in accordance with the Helsinki II Declaration of 1964, as revised in Edinburgh in 2000.

### Design and randomization

All participants were randomly allocated to receive infusion of 1.5 μg/min h-αCGRP (Calbiochem–Merck4Biosciences) or placebo (isotonic saline) over 20 min and scanned on two study days separated by at least 1 week. The CGRP dose is known to induce headache in volunteers without affecting the mean arterial blood pressure [19]. On both experimental days, the participants received sumatriptan (Imigran^®^ injection, Glaxo Wellcome Operations, Bernard Castle, UK) 6 mg subcutaneous injection 42 min after start of infusion. The first part of the study investigated changes in arterial circumference of middle meningeal and middle cerebral arteries in response to CGRP and sumatriptan by MR-angiography [14].

## Experimental procedures

All participants reported headache free to the laboratory. Coffee, tea, cocoa or other methylxanthine-containing foods or beverages, and tobacco were not allowed for at least 12 h before start of the study. Subjects were placed in the supine position in MR scan room and a venous catheter (Venflon^®^) was inserted into the left antecubital vein for infusion. We collected blood samples to determine the baseline hematocrit, potassium and sodium. The subjects were monitored with ECG, end-tidal CO_2_ (capnograph, Datex, Finland), blood oxygen saturation, blood pressure and heart rate (Veris monitor, Medrad, USA).

**Fig. 1:**
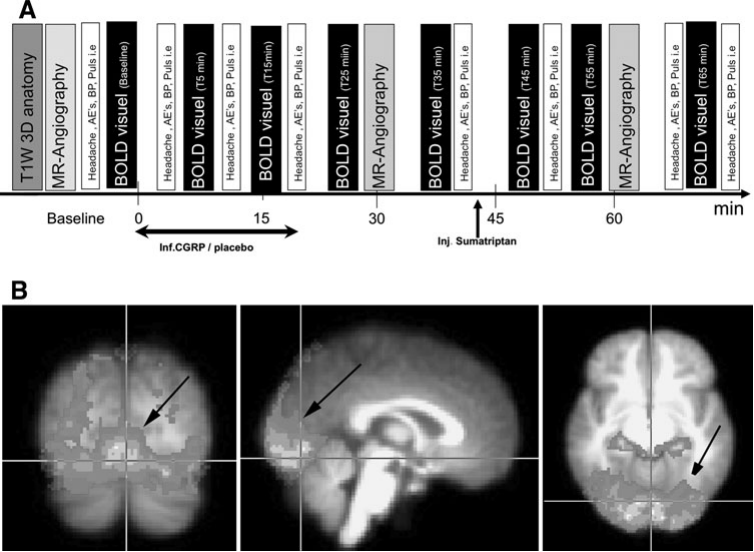
**a** The experimental paradigm. Baseline recordings were performed at *T*_−5min_. All participants randomly received infusion of CGRP (1.5 g/min) or placebo over 20 min. Injection of sumatriptan was given at *T*_42min_. T1-weighted 3D anatomical images were obtained at *T*_−15min_. BOLD-fMRI recordings were performed at baseline, *T*_5min_, *T*_15min_, *T*_25min_, *T*_35min_, *T*_45min_, *T*_55min_ and *T*_65min_. MR-angiography’s of the middle meningeal artery and middle cerebral artery were recorded at baseline, *T*_30min_ and *T*_60min_. Headache scores, blood pressure (BP), heart rate, end-tidal ${\text{P}}_{{{\text{CO}}_{ 2} }}$ and adverse events (AEs) were recorded at baseline, *T*_3min_, *T*_10min_, *T*_20min_, *T*_40min_, *T*_50min_, *T*_70min_ and *T*_75min_. **b** Group analysis of the baseline scans (before drug infusion) of 18 healthy subjects on CGRP study day. The images show at strong activation in the visual cortex after visual stimulation (*arrows*). Group analysis from the placebo day showed similar results

MR imaging was performed on a 3.0 T Philips Achieva Scanner (Philips Medical Systems, Best, The Netherlands) using an eight-element phased-array receive head coil. We first obtained a reference anatomical whole-brain image and then repeatedly measured the BOLD response after visual stimulation with a reversing checkerboard. We defined time of drug administration as *T*_0_. The anatomical image was recorded at −15 min, BOLD response at −5 min and at 5, 15, 25, 40, 50, 60 and 75 min. Headache intensity was recorded on a verbal rating scale (VRS) from 0 to 10 [0: no headache; 1: a very mild headache (including a feeling of pressing or throbbing); 10: worst imaginable headache] [20]. All variables were recorded at fixed time points throughout the study (Fig. 1a). Baseline was defined as before start of infusion (*T*_−5min_), the infusion phase lasted from 0 to 40 min where recordings during the infusion (0–20 min) and after the infusion were performed. The period after sumatriptan injection was defined as the sumatriptan phase.

## Data acquisition and imaging protocols

### Anatomical Images

Anatomical images were acquired using a T1-weighted 3D turbo field echo sequence (170 sagittal slices 1 mm thick; in-plane resolution 1 × 1 mm: repetition time 9.9 s; echo time 4.6 ms; flip angle 8o).

### BOLD response

BOLD functional imaging utilized a gradient echo EPI sequence (32 slices 4.0 mm thick; slice gap 0.1 mm; field of view 230 × 230 mm; in-plane acquired resolution 2.9 × 2.9 mm; repetition time 3.0 s; echo time 35 ms; flip angle 90°; SENSE factor 2). Slices were oriented parallel with the inferior border of corpus callosum covering the whole brain. The first four volumes of each run were discarded to avoid saturation effects. We obtained 100 volumes during each 5 min scan session.

To record the BOLD response, we applied visual stimulation with a checkerboard. This is a well-established modality that produces a rather large BOLD response in the visual cortex. Accordingly, we choose the visual cortex (V1, V2 and V3) as our region-of-interest (ROI). Visual stimulation was presented with the Eloquence system (Invivo, Orlando, Florida), using a pair of NNL goggles (NordicNeuroLab, Bergen, Norway). A fiber-optic cable connected the system to a control computer outside the scanner room. The paradigm consisted of rest blocks, where a uniform gray image was shown, alternating with active blocks displaying a black and white checkerboard reversing at 8 Hz. The block length was 1 min and two activation periods were included during a scan session which had a duration of 5 min. Subjects were asked to fixate on a central fixation cross during the entire scan. The onset of visual stimuli was triggered by the scan acquisition. Eye tracking (NordicNeuroLab, Bergen, Norway) was used to monitor their level of fixation.

## Data analysis and statistics

### BOLD data

Functional images were analyzed using FMRIB Software Library (FSL) version 5.98, Oxford, UK (http://www.fmrib.ox.ac.uk/fsl). FMRI Expert Analysis Tool (FEAT, version 5.98) was used for pre-processing (first level analysis). Pre-processing steps included motion-correction, brain extraction, and spatial (4 mm smoothing) and temporal filtering (high pass 200 s). A full quality assurance (QA) was done prior to the statistical analysis. Scans with severe distortions and/or excessive motion (>3 mm) were excluded from further analysis. Those that passed QA were included in the following statistical analysis. Statistical results were co-registered first to the subject’s own T1-weighted 3D anatomical images and subsequently to a standard Montreal Neurological Institute (MNI-152) atlas. The 3D anatomical images were transformed to match the dimensions of the functional scans using FSLSWAP and brain extraction was performed using the FSL Brain Extraction Tool (BET) (fractional intensity threshold: 0.6, threshold gradient of −0.1, and robust brain center estimation). For registration to the 3D anatomical images, linear registration, full search and nine degrees of freedom (DOF) were used, whereas 12 DOF was used for the subsequent registration to the standard MNI-152 atlas. The visual block stimulation paradigm convolved with a two gamma hemodynamic response function served as a model time course. *Z* (Gaussianised T/F) statistical images were thresholded using clusters determined by *Z* > 2.3 and a (corrected) cluster significance threshold of *P* = 0.05.

The BOLD response to visual stimulation was extracted both as ROI expressed as COPE1 values (contrast of parameter estimates) and voxel-wise. For the voxel-wise analysis, the FEAT tool was used (FEAT FSL version 4.1.6, Oxford for Mac). For the ROI analysis, the visual cortex (V1, V2 and V3) was identified based on the Juelich Histological Atlas and normalized to the MNI structural atlas (Feat query FSL, version 4.1.6, Oxford for Mac). The extracted values were then transferred to SPSS 18.0 for Mac (IBM SPSS, New York, USA) and baseline was corrected before further statistical analysis.

### ANOVA analysis of the effect and time and drug

All values are presented as mean ± SD and hemodynamic peak responses as mean percentage from baseline [95% confidence interval (CI)] except vascular data (blood pressure, heart rate, end-tidal ${\text{P}}_{{{\text{CO}}_{ 2} }}$, oxygen saturation), which are presented as mean ± SEM.

The primary end-points were changes over time in relative BOLD response after infusion of CGRP or placebo, difference in BOLD response between two experimental days, difference in BOLD response before and after sumatriptan administration and between experimental days.

We analyzed for changes over time for each experimental day separately with analysis of variance (ANOVA) with the fixed factors subjects and time. To reduce mass significance, the following time points were selected for analysis (*T*_−5_, *T*_5_, *T*_15_, *T*_25_ and *T*_40_). Baseline was defined as *T*_−5_. A second level analysis was performed to test CGRP versus placebo using a two-way ANOVA with the fixed factors time and drug. The sumatriptan phase of the CGRP day and the placebo day was analyzed in a similar way. Analysis for changes over time was performed for each day separately using ANOVA with subjects and time as fixed factors. The measured time points *T*_50_, *T*_60_ and *T*_75_ was compared against the functional scan immediately preceding sumatriptan administration (*T*_40_). A second level analysis was performed to compare the two study days.

We tested for period and carry-over effects for baseline hemodynamic variables using independent *t* test. Five percent (*P* < 0.05) was accepted as the level of significance.

## Results

All participants completed the study on both study days. Two subjects did not complete one scanning (5 min) each due to temporary scanner breakdown. One scan from two subjects had to be removed due to movement artefacts. There was no carry-over or period effect for BOLD response, blood pressure or heart rate (*P* > 0.05). Baseline blood samples showed normal hematocrit-, potassium- and sodium levels. Blood pressure, heart rate, oxygen saturation, end-tidal ${\text{P}}_{{{\text{CO}}_{ 2} }}$ did not change significantly during the experiment (*P* > 0.05) (for details see supplementary Table 1). All subjects showed a strong BOLD response to visual stimulation. Group activation is shown in Fig. 1b. Fourteen (77%) out of 18 participants reported CGRP induced immediate headache during the observation period of 0–42 min. Five participants reported headache on placebo day.

### The effect of exogenous CGRP on the BOLD response in the visual cortex

**Fig. 2:**
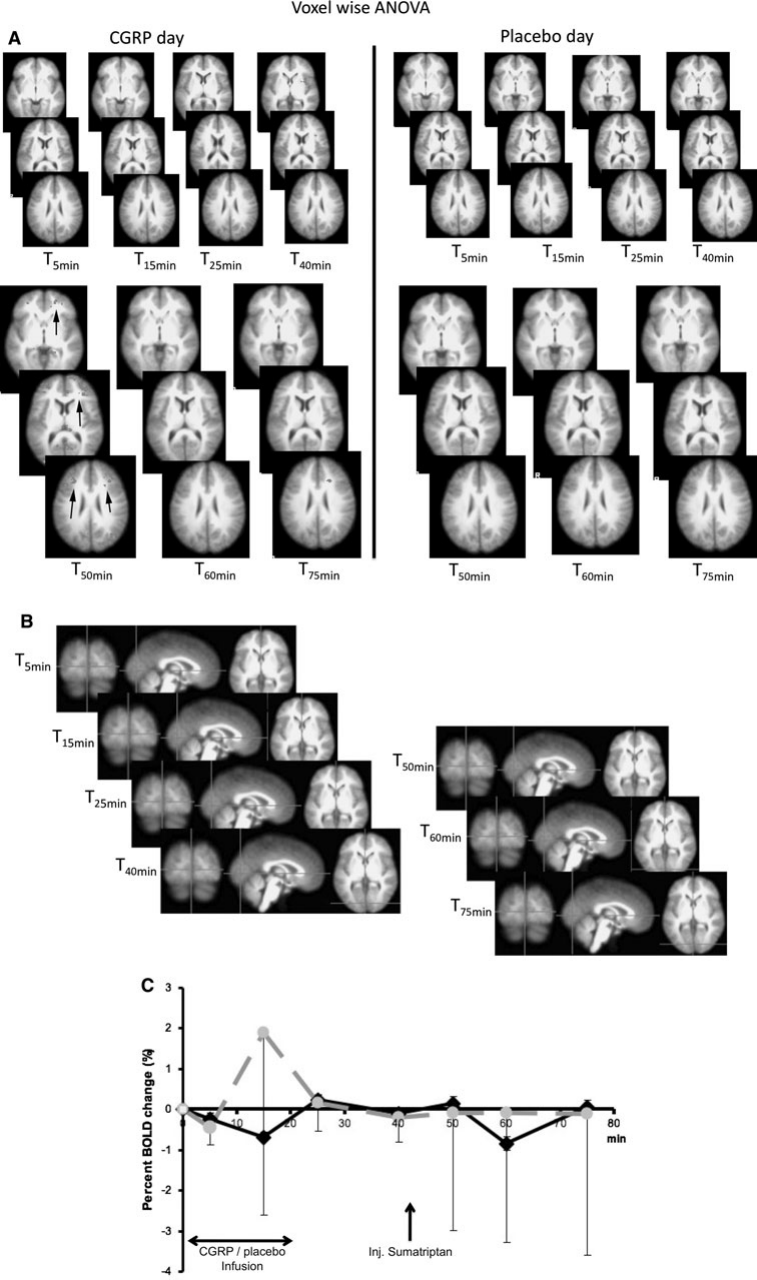
**a** One-way voxel-wise ANOVA results from the measured time points at the CGRP day (*left side*) and placebo day (*right side*). ANOVA showed no statistical changes over time in activated voxels after CGRP and placebo infusion except some scattered activation after sumatriptan injection on CGRP day. The activated voxels were located primarily in the cerebellum and in the white matter of the corpus callosum (*arrows*). There were no activated voxels in the predefined visual region-of-interest. **b** Second level analysis revealed no statistical difference between two experimental days. The pictures show the mean subtracted values between placebo day and the CGRP day. **c** Baseline corrected contrast of parameter estimate (COPE1) results from the visual region-of-interest. ANOVA revealed no statistical changes after CGRP (*P* = 0.12) or placebo infusion (*P* = 0.41). We found no statistical difference between two experimental days (*P* = 0.357). ANOVA revealed no statistical changes after injection of sumatriptan either on the CGRP (*P* = 0.71) or on the placebo days (*P* = 0.98). We found no statistical after sumatriptan between two experimental days (*P* = 0.49)

ANOVA did not show significant changes over time in activated voxels either on the CGRP day or on the placebo day (Fig. 2a, b). ANOVA of the COPE recordings in the visual cortex revealed no statistical changes after CGRP (*P* = 0.12) or placebo infusion (*P* = 0.41). We found no difference with regard to activated voxels or recordings of COPE1 values between the two experimental days (*P* = 0.357) (Fig. 2c).

### The effect of sumatriptan on the BOLD response in the visual cortex

On the CGRP day, one-way voxel-wise ANOVA after sumatriptan administration showed scattered activation at *T*_45min_. The activated voxels were located primarily in the cerebellum and in the white matter of the corpus callosum. There were no activated voxels in the visual cortex that was the predefined ROI or in other areas directly related to visual stimulation. The remaining recordings did not show significantly activated voxels. On the placebo day, one-way voxel-wise ANOVA revealed no significantly activated voxels (Fig. 2a, b). One-way ANOVA of the COPE1 recording values after sumatriptan administration revealed no statistical changes either on the CGRP day (*P* = 0.71) or on the placebo day (*P* = 0.98). We found no difference in activated voxels or recordings of COPE1 values between two experimental days (*P* = 0.49) (Fig. 2c).

## Discussion

The present study investigates for the first time the effect of intravenous CGRP and sumatriptan on brain activity using fMRI. Systemic administration of CGRP and sumatriptan caused *no* changes in neuronal activity of the visual cortex. Before discussing the main results, we shall briefly clarify some important issues regarding methods applied in the present study. When interpreting pharmacological fMRI data, it is important to keep in mind that changes in brain hemodynamics such as CBF, CBV and CMRO_2_ can alter the measured BOLD signal [21]. The BOLD signal is an indirect way of measuring brain activation. During brain activation, the increased supply of oxyhemoglobin results in a relative decrease in deoxyhemoglobin. While deoxy-hemoglobin is paramagnetic and oxyhemoglobin is diamagnetic, brain activation results in an increased MR signal as detected by appropriate MR techniques. CGRP is a very potent vasodilator and may theoretically affect the BOLD response. Therefore, as a first step to quantifying altered brain activity we investigated the effect of CGRP on the BOLD response in healthy volunteers. Experiments in healthy volunteers are usually a precondition for later studies in pathological conditions. We furthermore considered it as a necessary step to study the BOLD response in the visual cortex for the following reasons: (1) Visual stimulation produces a large BOLD signal in the visual cortex and, therefore, possible effects of CGRP on the BOLD response would be easy to quantify; (2) Before investigating possible specific effects of CGRP in the trigeminal pain pathways, it is important to determine if possible modulation of the BOLD signal is due to specific changes in these pathways or accounted to a general effect of CGRP on the BOLD signal. (3) The visual cortex is considered as an important compartment in migraine research [22] and before investigating possible effect of exogenous CGRP on excitability of visual cortex during headache in migraineurs, it is important to determine its effect in healthy volunteers.

### Cerebral effect of CGRP

The most important question raised by CGRP provocation experiments is whether intravenous infusion of CGRP induces headache inside or outside of the blood–brain barrier (BBB). The BBB is formed by the tight junctions between endothelial cells in cerebral vessels and the vascular smooth muscle cells are placed inside the BBB. Studies of cerebral arterial diameter or cerebral blood flow may, therefore, elucidate mechanisms underlying the effects of exogenous CGRP on the brain, when given systemically. Experimental studies in healthy volunteers [23] and migraine sufferers [24] reported no effect of intravenous infusion of CGRP on global or regional CBF. In vitro studies have shown that intraluminal CGRP did not dilate cerebral arteries but extraluminal application of CGRP did [13]. Together these studies indicate that exogenous CGRP does *not* cross the BBB in cerebral arteries.

CGRP is found in many regions of the CNS including visual cortex [12]. Interestingly, CGRP receptors are not detected in central glial cells or second order neurons [25]. It has been reported that CGRP facilitates glutamatergic neurotransmission in the dorsal horn of the spinal cord [26]. Furthermore, CGRP may modulate central sensitization by increasing the discharge frequency of wide dynamic range neurons in the spinal cord, thus modulating nociceptive transmission [27]. Interestingly, two studies have found that CGRP release is anti-nociceptive [28, 29].

### Extracerebral effect of CGRP

**Fig. 3:**
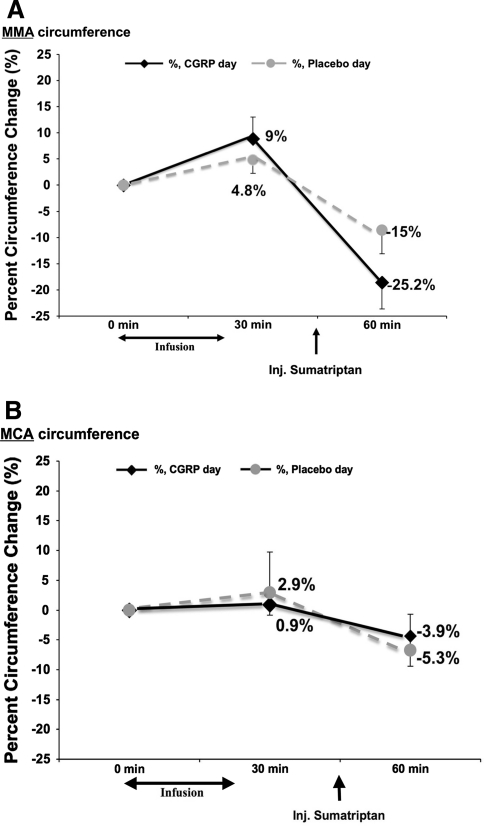
*Top* middle meningeal artery (MMA) circumference changes between baseline, after infusion of h-αCGRP or placebo and after subcutaneous sumatriptan 6 mg in 18 healthy subjects. There was significant 9% dilatation of MMA after CGRP compared to placebo (*P* = 0.006). Sumatriptan contracted the MMA by 25.2% (19.45–30.54%) on the CGRP day and 15% (7.66–22.34% CI) on the placebo day. *Bottom* middle cerebral artery (*MCA*) circumference changes between baseline, after infusion and after sumatriptan administration. MCA circumference did not differ between CGRP and placebo (*P* > 0.05). Sumatriptan contracted MCA by 3.9% (1.23–6.60% CI) on the CGRP day and by 5.3% (2.34–8.27% CI) after placebo. *Y* axis shows relative (%) changes compared to baseline while the present (%) values show relative changes between two measurements (baseline vs. infusion phase or infusion phase vs. after sumatriptan administration). *Error bars* indicate one-sided SEM. Reproduced with permission* from Asghar et al. (Neurology 2010;75(17):1520–1526) (*Permission has been requested)

CGRP is one of the most powerful vasodilators [30] and its receptor components are found in the smooth muscle cells of cranial arteries [31, 32]. In arteries without a BBB such as the MMA, exogenous CGRP reaches the smooth muscle cells and dilates the artery [32]. Petersen et al. [13] demonstrated that the extracerebral artery MMA relaxed after intraluminal administration of CGRP. In vitro studies of human [33, 34] and rat [35] MMA reported similar results. Previous human experiments showed 30% dilatation of the superficial temporal artery after CGRP infusion [8]. In the present study, we recorded BOLD-fMRI data and MRA data simultaneously. Angiography results of middle meningeal artery (MMA) and middle cerebral artery (MCA) have been previously presented [14]. We found that CGRP infusion induced a dilatation of MMA of 9.2%, but failed to induce diameter changes in the MCA or to alter baseline BOLD signal (see Fig. 3). Thus, CGRP acts outside of the BBB.

### Effect of sumatriptan on brain activity

The mechanism of anti-migraine action of triptans remains a matter of intense research and debate [36]. Sumatriptan (HT1B/1D receptor agonist) was originally developed as a selective cranial vasoconstrictor [37]. Electrophysiological studies on animals reported peripheral [36, 38] and central sites of action in the trigeminal pain pathway [39]. Sakai et al. [40] reported that the increased brain 5-HT synthetic rate during a migraine attack was reversed by sumatriptan. It is unclear, however, whether this was due to a direct or indirect effect of sumatriptan on the brain. In the present study, sumatriptan did not affect visual cortex activity or diameter of the MCA. However, sumatriptan reversed CGRP-induced dilatation of the MMA in healthy volunteers [14] and in migraine patients during CGRP-induced migraine [9]. These data suggest that sumatriptan does not cross the BBB but acts outside of the BBB possibly by contracting the MMA.

## Conclusion

Systemic administration of CGRP or sumatriptan has no direct effects on the BOLD signal in healthy volunteers. Given systemically, both migraine provoking peptide CGRP and anti-migraine drug sumatriptan do not modulate BOLD responses in the visual cortex.

*Clinical trial registration* The Ethical Committee of Copenhagen (H-KA-20060083) approved the study.

## Electronic supplementary material

Below is the link to the electronic supplementary material.

Supplementary material 1 (DOC 79 kb)
