# Supplementary material for: Effect of CGRP and sumatriptan on the BOLD response in visual cortex
Source: J Headache Pain. 2012 Jan 14;13(2):159–66. doi: 10.1007/s10194-011-0415-4 (PMC3274575; doi:10.1007/s10194-011-0415-4)
Supplement: Supplementary file 1 — Supplementary material 1 (DOC 79 kb) [file 10194_2011_415_MOESM1_ESM.doc]

**Supplementary Table 1**

Mean (± SEM) end-tidal P_CO2_, blood pressure, heart rate and O_2_ saturation during and after infusion of CGRP and placebo.

|  | **CGRP day** | | | | |
| --- | --- | --- | --- | --- | --- |
|  | P_CO2_ | Blood pressure | | Heart rate | O2 saturation |
|  |  | Systolic | Diastolic |  |  |
| Baseline | 5.21 (±0.072) | 118.6 (±2.07) | 66.93 (±1,83) | 64.51 (±2.7) | 98.62 (±0.30) |
| T_3min_ | 5.22 (±0.073) | 116.3 (±1.92) | 66.94 (±1.97) | 65.53 (±2.5) | 98.73 (±0.33) |
| T_10min_ | 5.33 (±0.084) | 118.4 (±2.07) | 66.88 (±1.76) | 69.41 (±2.4) | 98.67 (±0.31) |
| T_20min_ | 5.18 (±0.083) | 115.1 (±2.12) | 63.94 (±1.84) | 73.43 (±3.1) | 98.83 (±0.42) |
| T_40min_ | 5.16 (±0.084) | 118.4 (±2.07) | 66.88 (±1.76) | 69.41 (±2.4) | 98.67 (±0.33) |
| T_50min_ | 5.17 (±0.078) | 126.2 (±2.08) | 79,18 (±1.82) | 70.12 (±2.9) | 98.67 (±0.31) |
| T_70min_ | 5.22 (±0.083) | 123.1 (±1.73) | 76.03 (±1.74) | 68.72 (±2.6) | 98.57 (±0.43) |
| T_75min_ | 5.16 (±0.072) | 124.1 (±1.53) | 75.06 (±1.68) | 67.94 (±2.6) | 98.57 (±0.37) |

|  | **Placebo day** | | | | |
| --- | --- | --- | --- | --- | --- |
|  | P_CO2_ | Blood pressure | | Heart rate | O2 saturation |
|  |  | Systolic | Diastolic |  |  |
| Baseline | 5.21 (±0.085) | 118.8 (±3.25) | 67.88 (±2.17) | 67.12 (±2.83) | 98.50 (±0.27) |
| T_3min_ | 5.24 (±0.099) | 116.4 (±2.52) | 67.65 (±2.09) | 66.94 (±2.94) | 98.63 (±0.38) |
| T_10min_ | 5.21 (±0.082) | 116.4 (±2.22) | 66.69 (±1.55) | 70.31 (±3.55) | 98.57 (±0.43) |
| T_20min_ | 5.26 (±0.090) | 116.4 (±2.51) | 69.29 (±2.21) | 66.47 (±2.93) | 98.63 (±0.38) |
| T_40min_ | 5.26 (±0.082) | 117.0 (±3.10) | 69.71 (±2.30) | 69.35 (±3.28) | 98.50 (±0.38) |
| T_50min_ | 5.24 (±0.080) | 125.4 (±2.46) | 82.12 (±2.15) | 69.53 (±2.78) | 98.75 (±0.16) |
| T_70min_ | 5.19 (±0.080) | 124.7 (±2.01) | 78.12 (±2.28) | 66.94 (±3.56) | 98.75 (±0.25) |
| T_75min_ | 5.21 (±0.063) | 124.5 (±2.34) | 76.41 (±2.24) | 65.12 (±2.60) | 98.87 (±0.13) |
